# The link between ankylosing spondylitis and oral health conditions: two nested case-control studies using data of the UK Biobank

**DOI:** 10.1590/1678-7757-2018-0207

**Published:** 2018-11-08

**Authors:** Hadeel Mohammed Abbood, Ejaz Pathan, George P. Cherukara

**Affiliations:** 1 University of Aberdeen, School of Medicine, Medical Sciences & Nutrition, Institute of Dentistry, Foresterhill Campus, Foresterhill, Scotland, United Kingdom; 2 College of Dentistry, University of Tikrit, Salahiddin, Iraq; 3 Toronto Western Hospital, University Health Network, Spondylitis Program, Toronto, Canada

**Keywords:** Ankylosing spondylitis, Oral health, Oral ulcers, Epidemiology

## Abstract

Ankylosing Spondylitis (AS) is an inflammatory rheumatic disease that affects the axial skeleton and the sacroiliac joints. Recent studies investigated the link between AS and oral diseases, particularly periodontitis. Others suggested that periodontitis may have a role in the pathogenesis of rheumatic diseases. Objective: The aim of this study is to investigate the association between AS and oral conditions. Material and Methods: This research was conducted using the UK Biobank Resource under Application Number 26307. The UK Biobank recruited around 500000 participants throughout Great Britain. Clinical records were available for 2734 participants. Two case-control studies were conducted based on whether AS was self-reported or clinically diagnosed. Oral conditions were identified using self-reported reports of oral ulcers, painful gums, bleeding gums, loose teeth, toothache, and dentures. The association between AS and oral conditions was assessed using logistic regression adjusted for age, gender, educational level, smoking status, alcohol consumption, and body mass index. Results: A total of 1307 cases and 491503 control participants were eligible for the self-reported AS study. The mean age was 58 years for the cases [7.5 standard deviation (SD)] and 57 years for the control groups (8.1 SD). Also, 37.1% of the cases and 54.2% of the control participants were females. Among the oral conditions, only oral ulcers were strongly associated with AS [1.57 adjusted odds ratio (OR); 95% confidence interval (CI) 1.31 to 1.88]. For the study of clinically diagnosed AS, 153 cases and 490351 control participants were identified. The mean age for both cases and control groups was 57 years; 7.6 SD for the cases and 8.1 for the control group. Females corresponded to 26.1% of the cases, and 54.2% of the control participants. Clinically diagnosed AS was associated with self-reported oral ulcers (2.17 adjusted OR; 95% CI 1.33 to 3.53). Conclusion: Self-reported and clinically diagnosed AS populations have increased risk of reporting oral ulcers. Further investigations are required to assess the link between a specific type of oral condition and AS.

## Introduction

Ankylosing Spondylitis (AS) is a type of chronic inflammatory rheumatic disease that affects the axial skeleton and the sacroiliac joints. Extra-articular manifestations might be associated as well, such as iritis, psoriasis, inflammatory bowel disease and genitourinary tract inflammation. ^1^

Recent studies investigated the link between AS and oral diseases, particularly periodontitis. ^2^ ^–^ ^4^ Generally, oral diseases are prevalent chronic diseases worldwide; especially periodontal disease and dental caries. Their impact on general health raises their importance. ^5^

Periodontal disease is a group of diseases which affect the tissue surrounding the teeth. It might lead to the destruction of the alveolar bone and loss of tooth support. These diseases are characterised by periodontal tissue inflammation, gingival bleeding, pocket formation, and/or tooth mobility. ^6^ Its prevalence and severity increase with age. ^7^ Periodontal diseases are initiated by periodontal pathogens. However, host response in conjunction with several risk factors, such as cigarette smoking and emotional stress, play an important role in the initiation and progression of periodontal destruction. ^7^

There is growing evidence that periodontitis is linked and might initiate other systemic conditions such as diabetes mellitus and cardiovascular disease. ^8^ Others hypothesised that periodontal pathogens, especially *Porphyromonas gingivalis,* could be the triggering factor for the autoimmunity against citrullinating proteins in the joints, which might initiate rheumatoid arthritis (RA). ^9^ Evidence of similarity in the immune-pathologic condition between AS and periodontitis is present. Significant elevations in the levels of interleukin 6 and tumour necrosis factor- alpha (TNF-α) are present in both AS and periodontitis patients. ^10^ ^,^ ^11^

Due to the difficulties in conducting population- based studies using clinical diagnosis, self-reported disease is an alternative way of diagnosis. Selfreported AS was shown to be agreeable with the clinical records ^12^ , whereas self-reported periodontal disease was shown to be agreeable with the clinical diagnosis of periodontitis. ^13^ It was also shown that the reporting of the number of natural teeth, presence or absence of prosthetic appliance, and root canal filling by the patients was agreeable with the clinical findings for these patients. ^14^

The aims of this study are:

1. To report the findings of two case-control studies based on the UK Biobank database;
2. Identify any associations between AS and oral conditions identified in the database.

## Material and methods

### UK Biobank

The UK Biobank is a large population-based cohort study with approximately half a million participants recruited from 22 assessment centres throughout Great Britain between 2006 and 2010. Their age ranged between 40 to 69 years at the time of recruitment. The baseline assessment includes electronic signed consent, self-completed touch-screen questionnaire, computer-assisted interview, physical and functional measures, and blood, urine and saliva sample collection. ^15^ ^,^ ^16^ For the present analysis, only baseline data was used.

The UK Biobank gained ethical approvals from the North West Multi-Centre Research Ethics Committee, the Community Health Index Advisory Group, the Patient Information Advisory Group, and the National Health Service National Research Ethics Service. Detailed cohort protocol, scientific rationale, and study design are available online. ^15^

### Study design

Two nested case-control studies were conducted based on the method for defining AS; self-reported AS case-control study, and clinically diagnosed AS case-control study. The first study investigated whether individuals who self-reported AS have higher prevalence of oral health problems than those who did not. The outcome is self-reported AS and the exposure is self-reported oral conditions. The second case-control study investigated the association between clinically diagnosed AS and self-reported oral conditions.

### Identifying AS cases

In the self-reported AS case-control study, AS was identified by asking the participants whether they had been told by a doctor that they have some sort of severe non-cancer illness or disability. If the participant was uncertain of the type of illness they had, they described it to the interviewer (a trained nurse) who attempted to place it within the coding tree. If the illness could not be located in the coding tree, then the interviewer entered a free-text description of it. These free-text descriptions were subsequently examined by a doctor and, where possible, matched to entries in the coding tree. Free-text descriptions which could not be matched with very high probability were marked as "unclassifiable"

In the clinical case-control study, AS was identified based on clinical records using the International Classification of Diseases, 10^th^ Revision (ICD-10). Clinical records were available for 2734 participants only.

### Definition of oral conditions

Oral conditions were defined according to the selfreported data on mouth/teeth or dental problems. The participants used a touchscreen to answer the question *"Do you have any of the following? (You can select more than one answer)”.* The possible answers were: Mouth ulcers, painful gums, bleeding gums, loose teeth, toothache, and dentures.

### Exclusion criteria

Self-reported RA, and other back problems which are not classified as AS such as spine arthritis/ spondylosis were excluded from the analysis plan of self-reported AS.

During the analysis of clinically diagnosed AS, the participants with clinically diagnosed RA and selfreported RA were excluded from both the cases and control groups. Similarly, self-reported AS participants were excluded from the control group only to avoid including participants who might have delayed clinical AS diagnosis.

### Data analysis

Descriptive analysis was used to describe the characteristics of the study population including total number and percentage. Mean and standard deviation (SD) were used to describe the age of the participants.

In order to assess the relationship between AS status and oral conditions, a logistic regression model was used to calculate the odds ratio (OR) and 95% confidence interval (CI) adjusted for age, gender, educational qualification level, smoking status, alcohol consumption, and body mass index. Statistical significance level was defined at p=0.05. All data were processed using the IBM SPSS Statistics Software Package, version 24.

## Results

As we have conducted two case-control studies, we will describe the findings of each study separately.

### Self-reported AS case-control study

#### Population description

Following the exclusion criteria, 1307 participants reported having AS and 491503 were considered as part of the non-AS or control groups. The mean age of the AS cases group was 58 years (SD 7.5), and 57 years for the control group(SD 8.1). The age group between 55 and 64 years old predominated both in the AS and non-AS groups (49.8% and 42%, respectively). Over a third of the AS (37.1%) and half of the control group (54.2%) were female. The education level for both AS and non-AS participants was roughly the same: 39.1% of the AS cases and 39.8% of the non-AS participants had college or university degree. Current smokers were more prevalent among the AS cases compared to the non-AS participants (14.9% and 10.5%, respectively). On the other hand, a lower percentage of AS participants reported having never smoked compared to the non-AS cases (44.6% *vs.* 55%). Please see Table 1 for more details.

**Table 1:** Socioeconomic description of the self-reported ankylosing spondylitis (AS) and control groups, excluding self-reported rheumatic disease

		Self-reported AS n. (%)	Non-AS n. (%)
Total number		1307	491503
Mean age (Standard deviation) in years		58.0 (7.5)	57.0 (8.1)
Age group	Less than 45	92 (7.0)	50695 (10.3)
	45 to 54	325 (24.9)	140010 (28.5)
	55 to 64	651 (49.8)	206477 (42.0)
	65 or older	238 (18.2)	93898 (19.1)
Gender	Female	485 (37.1)	266459 (54.2)
	Male	822 (62.9)	225044 (45.8)
Qualifications	College or University degree	398 (39.1)	158890 (39.8)
	A levels/AS levels or equivalent	117 (11.5)	54321 (13.6)
	O levels/GCSEs or equivalent	274 (26.9)	102919 (25.8)
	CSEs or equivalent	56 (5.5)	26348 (6.6)
	NVQ or HND or HNC or equivalent	99 (9.7)	31931 (8.0)
	Other professional qualifications e.g. nursing, teaching.	75 (7.4)	25144 (6.3)
Smoking status	Never	579 (44.6)	268519 (55.0)
	Previous	527 (40.6)	168669 (34.5)
	Current	193 (14.9)	51451 (10.5)
Alcohol intake frequency	Daily or almost daily	300 (23.0)	100014 (20.4)
	3 or 4 times a week	274 (21.0)	113507 (23.2)
	Once or twice a week	327 (25.1)	126601 (25.8)
	1 to 3 times a month	127 (9.7)	54547 (11.1)
	Special occasions only	165 (12.6)	56189 (11.5)
	Never	112 (8.6)	39167 (8.0)
Body Mass index	Normal weight or underweight	380 (29.6)	161268 (33.1)
	Overweight	581 (45.3)	207445 (42.6)
	Obese	321 (25.0)	118744 (24.4)

### Prevalence of oral conditions and their association with self-reported AS

Generally, the AS participants reported a higher prevalence of oral conditions compared to the control group ( Table 2 ). Also, they reported a higher prevalence of oral ulcers than the control group (14.5% *vs.* 9.9%). There was more than 50% increase in the risk of reporting AS among those who reported having oral ulcers (1.54 crude OR; 95% CI 1.32 to 1.80). Furthermore, no change was observed after adjusting it for confounding factors (1.57 adjusted OR; 95% CI 1.31 to 1.88). Further analysis was conducted by excluding participants who reported having dentures, to exclude oral conditions related to tooth loss and denture wear ( Table 3 ). By doing this, no change was shown in the estimated risk (1.48 crude OR; 95% CI 1.24 to 1.77, and 1.48 adjusted OR; 95% CI 1.21 to 1.80).

**Table 2:** Prevalence of self-reported oral health conditions in self-reported AS and non-AS populations

Oral condition	AS cases n. (%) (n=1307)	Non-As n. (%) (n=491503)	Crude OR (95% CI)	Adjusted OR (95% CI)
Oral ulcers	189 (14.5)	48612 (9.9)	1.54 (1.32-1.80)	1.57 (1.31-1.88)
Painful gums	61 (4.7)	14685 (3.0)	1.59 (1.23-2.06)	1.34 (0.96-1.86)
Bleeding gums	117 (13.5)	64903 (13.2)	1.03 (0.88-1.21)	1.08 (0.90-1.30)
Loose teeth	81 (6.2)	21053 (4.3)	1.48 (1.18-1.85)	1.24 (0.94-1.63)
Toothache	75 (5.7)	21637 (4.4)	1.32 (1.05-1.67)	1.09 (0.82-1.45)
Dentures	290 (22.2)	80276 (16.3)	1.46 (1.28-1.67)	1.33 (1.13-1.58)

OR=Odds ratio, CI=Confidence interval, Adjusted OR=Adjusted for age, gender, education level, smoking status, alcohol frequency intake, and body mass index

**Table 3:** Prevalence of self-reported oral health condition in self-reported AS and non-AS populations after excluding those who reported having dentures

Oral condition	AS cases n. (%) (n=1017)	Non-As n. (%) (n=411227)	Crude OR (95% CI)	Adjusted OR (95% CI)
Oral ulcers	145 (14.3)	41457 (10.1)	1.48 (1.24-1.77)	1.48 (1.21-1.80)
Painful gums	43 (4.2)	11604 (2.8)	1.52 (1.12-2.07)	1.18 (0.79-1.76)
Bleeding gums	144 (14.2)	58157 (14.1)	1.00 (0.84-1.20)	1.05 (0.86-1.29)
Loose teeth	50 (4.9)	14735 (3.6)	1.39 (1.05-1.85)	1.12 (0.79-1.59)
Toothache	57 (5.6)	19039 (4.6)	1.22 (0.94-1.60)	0.95 (0.68-1.32)

OR=Odds ratio, CI=Confidence interval, Adjusted OR=Adjusted for age, gender, education level, smoking status, alcohol frequency intake, and body mass index

The prevalence of painful gums was relatively higher in the AS cases (4.7%) than in the control group (3%). Although a statistically significant association was shown between AS and painful gums (1.59 crude OR; 95% CI 1.23 to 2.06), adjusting it for confounding factors changed the association to non-significant (1.34 adjusted OR; 95% CI 0.96 to 1.86). No change was shown in OR after excluding participants with dentures ( Tables 2 and 3 ).

Similarly, the same prevalence of bleeding gums was shown in both AS and non-AS participants (13.5% and 13.2%, respectively). No statistically significant association was found between bleeding gums and self-reported AS (1.03 crude OR; 95% CI 0.88 to 1.21, 1.08 adjusted OR; 95% CI 0.90 to 1.30). Excluding participants who reported having dentures did not show any significant change in OR ( Tables 2 and 3 ).

In relation to loose teeth, 6.2% of the AS participants and 4.3% of the non-AS participants reported it. The logistic regression model showed an association between reporting loose teeth and increased risk of reporting AS (1.48 crude OR; 95% CI 1.18 to 1.85). However, this association became non-significant once adjusted for confounding factors (1.24 adjusted OR; 95% CI 0.94 to 1.63). Although participants with dentures were excluded, the OR results were relatively the same between the two regression models (1.39 crude OR; 95% CI 1.05 to 1.85, 1.12 adjusted OR; 95% CI 0.79 to 1.59).

The AS participants reported higher prevalence of toothache (5.7%) in relation to the non-AS participants (4.4%). Although the difference between both proportions was not relatively high, there was 32% increase in the risk of reporting AS with the increase in toothache prevalence (1.32 crude OR; 95% CI 1.05 to 1.67). However, adjusting this association for confounding factors made it non-statistically significant (1.09 adjusted OR; 95% CI 0.82 to 1.45). Excluding participants with dentures did not show any significant change in this association (1.22 crude OR; 95% CI 0.94 to 1.60, 0.95 adjusted OR; 95% CI 0.68 to 1.32) ( Tables 2 and 3 ).

Around one fourth of the AS participants reported having dentures compared to 16.3% of the non-AS participants. Denture wear resulted in a 46% increase in the risk of reporting AS (1.46 crude OR; 95% CI 1.28 to 1.67). This statistically significant association persisted after adjusting it for confounding factors (1.33 adjusted OR; 95% CI 1.13 to 1.58) ( Table 2 ).

### Clinical AS case-control study

#### Population description

In this study, 153 participants clinically diagnosed with AS and 490351 serving as control were identified after excluding clinically diagnosed RA, self-reported AS (from the control group only), and self-reported RA. The mean age was the same for both AS and non-AS participants (57 years; 7.6 SD and 8.1 SD for AS and non-AS, respectively). The distribution of age group between AS and non-AS participants was roughly the same: 9.2% of AS and 10.3% of non-AS participants had less than 45 years of age, and 16.3% of the AS and 19.1% of the non-AS participants were older than 64. Females consisted of around a quarter of the AS cases (26.1%), and half of the non-AS participants (54.2%). In broad terms, the distribution of educational qualification level in the AS and non-AS participants was roughly the same. The number of AS participants who had a college or university degree was relatively lower than the number for non-AS participants (35.7% and 39.8%, respectively) ( Table 4 ).

**Table 4:** Socioeconomic description of the clinically diagnosed AS cases and control groups, excluding clinically diagnosed rheumatoid arthritis (RA), self-reported AS in the control group only, and self-reported RA

		Clinically diagnosed AS n. (%)(n=153)	Non-AS n. (%) (n=490351)
Mean Age (Standard deviation) in years		57.0 (7.6)	57.0 (8.1)
Age group	Less than 45	14 (9.2)	50651 (10.3)
	45 to 54	41 (26.8)	139857 (28.5)
	55 to 64	73 (47.7)	206137 (42.0)
	65 or older	25 (16.3)	93706 (19.1)
Gender	Female	40 (26.1)	265957 (54.2)
	Male	113 (73.9)	224817 (45.8)
Qualifications	College or University degree	41 (35.7)	158723 (39.8)
	A levels/AS levels or equivalent	17 (14.8)	54266 (13.6)
	O levels/GCSEs or equivalent	26 (22.6)	102779 (25.8)
	CSEs or equivalent	12 (10.4)	26307 (6.6)
	NVQ or HND or HNC or equivalent	9 (7.8)	31882 (8.0)
	Other professional qualifications e.g.: nursing, teaching	10 (8.7)	25107 (6.3)
Smoking status	Never	60 (39.5)	268188 (55.0)
	Previous	68 (44.7)	168370 (34.5)
	Current	24 (15.8)	51365 (10.5)
Alcohol intake frequency	Daily or almost daily	24 (15.7)	99901 (20.4)
	3 or 4 times a week	28 (18.3)	113384 (23.2)
	Once or twice a week	50 (32.7)	126430 (25.8)
	1 to 3 times a month	11 (7.2)	54472 (11.1)
	Special occasions only	27 (17.6)	56046 (11.5)
	Never	13 (8.5)	39065 (8.0)
Body Mass index	Normal weight or underweight	39 (27.5)	161072 (33.1)
	Overweight	53 (37.3)	207149(42.6)
	Obese	50 (35.2)	118520 (24.3)

### Prevalence of oral conditions and their association with clinically diagnosed AS

From the general overview of Tables 5 and 6 , the prevalence of oral conditions was relatively higher in the AS participants compared to the non-AS participants, apart from the prevalence of bleeding gums. The prevalence of oral ulcers was significantly higher in the AS cases (15.7%) than in the non-AS participants (9.9%). The risk of AS increased with the increase in reporting oral ulcers (1.70 crude OR; 95% CI 1.01 to 2.62). Additionally, OR increased to 2.17 when adjusted for confounding factors; 95% CI 1.33 to 3.53. Excluding participants with dentures increased the prevalence of oral ulcers in the AS cases as well as in the non-AS participants, with higher prevalence in the former (17.7% and 10.1%, respectively). The risk of AS increased as well (1.91 crude OR; 95% CI 1.19 to 3.06, and 2.41 adjusted OR; 95% CI 1.43 to 4.06).

**Table 5:** Prevalence of self-reported oral conditions in the clinically diagnosed AS cases and control groups

Oral condition	AS cases n. (%) (n=153)	Non-As n. (%) (n=490351)	Crude OR (95% CI)	Adjusted OR (95% CI)
Oral ulcers	24 (15.7)	48514 (9.9)	1.70 (1.01-2.62)	2.17 (1.33-3.53)
Painful gums	8 (5.2)	14654 (3.0)	1.79 (0.88-3.65)	1.59 (0.65-3.91)
Bleeding gums	19 (12.4)	64813 (13.2)	0.93 (0.58-1.51)	0.98 (0.56-1.73)
Loose teeth	10 (6.5)	21021 (4.3)	1.56 (0.82-2.97)	1.34 (0.62-2.91)
Toothache	14 (9.2)	21604 (4.4)	2.19 (1.26-3.79)	1.27 (0.59-2.74)
Dentures	34 (22.2)	80099 (16.3)	1.47 (1.00-2.14)	1.31 (0.78-2.18)

OR=Odds ratio, CI=Confidence interval, Adjusted OR=Adjusted for age, gender, education level, smoking status, alcohol frequency intake, and body mass index

**Table 6:** Prevalence of self-reported oral conditions in the clinically diagnosed AS cases and control groups, excluding participants who reported having dentures

Oral condition	AS cases n. (%) (n=119)	Non-As n. (%) (n=410252)	Crude OR (95% CI)	Adjusted OR (95% CI)
Oral ulcers	21 (17.7)	41381 (10.1)	1.91 (1.19-3.06)	2.41 (1.43-4.06)
Painful gums	5 (4.2)	11582 (2.8)	1.51 (0.62-3.70)	1.68 (0.62-4.60)
Bleeding gums	13 (10.9)	58078 (14.1)	0.75 (0.42-1.33)	0.71 (0.35-1.42)
Loose teeth	7 (5.9)	14715 (3.6)	1.68 (0.78-3.61)	1.39 (0.56-3.47)
Toothache	12 (10.1)	19009 (4.6)	2.31 (1.27-4.20)	1.07 (0.43-2.64)

OR=Odds ratio, CI=Confidence interval, Adjusted OR=Adjusted for age, gender, education level, smoking status, alcohol frequency intake, and body mass index

Although the prevalence of painful gums in the AS cases was higher than in the non-AS participants (5.2% vs. 3%), no statistically significant association was found between AS and painful gums (1.79 crude OR; 95% CI 0.88 to 3.65, and 1.59 adjusted OR; 95% CI 0.65 to 3.91). Excluding participants with dentures showed no statistically significant changes (1.51 crude OR; 95% CI 0.62 to 3.7, and 1.68 adjusted OR; 95% CI 0.62 to 4.60).

The prevalence of bleeding gums was roughly the same in the AS cases and in the non-AS participants (12.4% and 13.2%, respectively). Consequently, there was no association found between AS and bleeding gums (0.93 crude OR; 95% CI 0.58 to 1.51, and 0.98 adjusted OR; 95% CI 0.56 to 1.73). Excluding participants using dentures led to a decrease in the prevalence of bleeding gums in the AS cases (10.9%), and to an increase in the non-AS participants (14.1%). However, these changes did not affect the association between AS and bleeding gums (0.75 crude OR; 95% CI 0.42 to 1.33, and 0.71 adjusted OR; 95% CI 0.35 to 1.42).

The AS cases had higher prevalence of loose teeth than the non-AS participants (6.5% vs. 4.3%). Nevertheless, no association was found between AS and loose teeth (1.56 crude OR, 95% CI 0.82 to 2.97, and 1.34 adjusted OR; 95% CI 0.62 to 2.91). Excluding participants with dentures decreased the prevalence of loose teeth in both groups, whilst maintaining the higher prevalence in the AS cases (5.9% in the AS cases and 3.6% in the non-AS participants). However, no association was shown between AS and loose teeth (1.68 crude OR, 95% CI 0.78 to 3.61, and 1.39 adjusted OR, 95% CI 0.56 to 3.47).

Of the AS participants, 9.2% reported having toothache, which was twice the value found for the non-AS cases (4.4%). Regression analysis showed a two-fold increase in the risk of AS with the increase in toothache reports (2.19 crude OR; 95% CI 1.26 to 3.79). However, this association faded when confounding factors were introduced into the regression model (1.27 adjusted OR; 95% 0.59 to 2.74). By excluding participants with dentures, the prevalence of toothache reports remained higher in the AS cases than in the non-AS participants (10.1% and 4.6%, respectively). Simultaneously, no significant changes were found in the results of the regression analysis (2.31 crude OR; 95% CI 1.27 to 4.20, and 1.07 adjusted OR; 95% CI 0.43 to 2.64).

The participants who reported having dentures represent nearly one fourth of the AS cases (22.2%), whereas only 16.3% of the non-AS participants reported having dentures. An association was found between AS and reporting having dentures (1.47 crude OR; 95% CI 1.00 to 2.14). However, taking confounding factors into account during the regression analysis led to a non-statistically significant association between AS and denture reports (1.31 adjusted OR; 95% CI 0.78 to 2.18).

## Discussion

Our findings demonstrate a link between AS and oral ulcers. Other oral conditions, such as painful gums, bleeding gums, loose teeth, toothache and reporting having dentures were not associated with AS; however, their prevalence was generally higher in the AS than in the control group.

### Method critique

Generally, the AS and control groups were not different in relation to mean age. This supports the homogeneity of the two populations. The AS population was significantly different from the non-AS population in terms of male/female ratio, smoking status, and education level. In the AS cases, the number of males was three times higher than the number of females. This gender ratio was previously shown globally, ^17^ as AS affects males more than females. Recently, the effect of smoking was shown as a risk factor for AS. ^18^ It has been shown that smoking might be associated with the development and progression of AS. This is in line with our study which showed a high rate of current smokers in the AS compared to the non-AS population. In the clinically diagnosed AS cases, those who had a higher education degree were less prevalent when compared to the non-AS population. These findings corroborate a previous study ^19^ and the relationship between socioeconomic factors and AS. The generalisability of the study is supported by the comparable demographics for the global AS population.

The UK Biobank has been designed for the purpose of investigating the exposure-disease association. These kinds of studies need a large number of participants with different levels of exposure in order to produce results that can be generalised to the population. The large heterogenic population included in the UK Biobank provide a valid interpretation for the exposure-disease association ^20^ which is a merit of this study.

Our study took common risk factors into account during the analysis, however, the identification of oral conditions was carried out through self-reported questions, not specific to any oral disease in particular. Using self-report to identify oral conditions as was done in this study might spoil the findings, however, due to issues related to non-feasibility of the clinical diagnosis, cost and time in large epidemiologic studies, the use of self-reported measures is justifiable. Selfreported oral conditions were found to be able to identify oral conditions when confirmed with a clinical diagnosis. ^14^ ^,^ ^21^

This being a nested case-control study, it was impossible to show the temporality in the association between any oral health conditions and AS diagnosis or self-report; therefore, the causal relationship could not be predicted. Moreover, as the UK Biobank's population was aged 40 to 69 at the recruitment stage, ^16^ the prevalence of oral conditions might be already high. For example, the rate of severe periodontitis is twice that of those aged between 30 and 44 in people aged 45 years and older. ^22^

### Association between AS and oral ulcers

Our findings showed a strong association between AS and oral ulcers. These findings were constant through all the analysis procedures including selfreported AS, clinically diagnosed AS, and when participants with dentures were excluded. Oral ulcer has various aetiologies including trauma, autoimmune, microbial infections, systemic disorders, and neoplasm. ^23^ Excluding participants with dentures during the analysis eliminates traumatic oral ulcers caused by denture wear. To the best of our knowledge, no study has investigated the association between AS and oral ulcers. Oral ulceration has been associated with other rheumatic diseases such as reactive arthritis, Behçet's disease, and systemic lupus erythematosus. ^24^ It could also be an adverse reaction to certain drugs, some of which are used in treating AS, such as non-steroidal anti-inflammatory drugs ^25^ and Methotrexate, ^26^ ^,^ ^27^ although the latter drug is not used in all AS patients. However, these findings were published in case reports; no population-based observational study or randomised control trial has been conducted to prove them. Unfortunately, we do not have sufficient information on the type of medication used by AS patients in this dataset. A study based on clinical examination of the oral ulcer and its time-related association with AS might be useful in identifying the pathogenesis behind it.

### Association between AS and other oral conditions

Bleeding gums, painful gums, toothache, and loss of natural teeth could all be shared manifestations and markers of highly prevalent oral diseases such as periodontitis and dental caries. ^5^ ^,^ ^6^ ^,^ ^28^ Several studies hypothesised the link between AS and periodontitis. ^2^ ^,^ ^29^ ^,^ ^30^ Nevertheless, there is disagreement regarding this association. ^2^ ^,^ ^4^ ^,^ ^31^ This disagreement derives from the difference in the case definition of periodontitis used by researchers, and the limited number of population-based studies conducted in this field. ^2^ Moreover, common risk factors between AS and periodontitis were not investigated thoroughly. ^2^

Despite the high prevalence of painful gums and loose teeth in AS cases compared to non-AS cases, our study did not find a significant association between these two parameters and AS. Although painful gums and loose teeth showed an association with selfreported AS, this association became non-significant once the analysis was adjusted for confounding factors.

Loose teeth is a sign of advanced stage of periodontitis. It is a consequence of alveolar bone destruction and the affected tooth's loss of periodontal support. Self-reported bleeding gums and loose teeth have been shown to corroborate the clinical findings of periodontitis. ^13^ Although self-reported painful gums showed high specificity (ability to recognise true negative findings), its sensitivity (ability to recognise true positive findings) was too low. ^13^ In the UK Biobank's data, oral health conditions were only reported in general without clarifying individual conditions. These factors might overestimate the significance of the association between oral conditions and AS. In addition, painful gums are not a common sign of chronic periodontitis, unless there is acute exacerbation. However, it might be a sign of gingivitis or necrotising periodontitis which are related diseases. Therefore, painful gums might not be the right measure to identify periodontitis.

To the best of our knowledge, no study has investigated the link between toothache and AS. However, one study showed an association between self-reported arthritis and toothache ^32^ which might oppose our findings. Toothache could result from acute periodontitis (i.e., periodontal abscess). ^28^ However, it is impossible to diagnose the actual cause of toothache based on the self-reported questionnaire only. Toothache has previously been shown to be related to poor oral health and thus to quality of life, as it affects the daily performance of the individual. ^33^ ^,^ ^34^ Therefore, associating toothache with arthritis could be a sign of low health-related quality of life.

Those who have dentures have lost their teeth partially completely. Our findings suggest that losing teeth increases the risk of AS, but these results were found only among the self-reported AS patients.

Losing natural teeth could be due to a range of causes, mostly including advanced periodontitis, dental caries, and trauma. It is impossible to predict the number and the cause of natural teeth loss by asking about the presence or absence of dentures. It is generally accepted that losing natural teeth can predict bad oral health and has an impact on the patients’ quality of life. ^35^ In addition, losing natural teeth is associated with age, which might explain the faded association with AS once common risk factors were considered in the analysis.

From a general look at the results of our study, the AS cases showed higher prevalence in the self-report of oral health conditions compared to the non-AS participants. This might draw the attention to the fact that the AS patients might have worse oral health compared to the control group, which may indicate need for health care organisations and authorities to pay more attention to this group of patients in order to increase their health-related quality of life in general and also their oral health-related quality of life.

## Conclusion

Within the limitations of this study, the AS population showed increased risk of reporting oral ulcers. There was an increase in the prevalence of reports of painful gums, toothache, loose teeth and loss of natural teeth in the AS compared to the non-AS population. However, when common risk factors between AS and oral conditions were taken into account, this association was impaired. Therefore, further investigations are required in this field by designing large population-based studies, with clear and concise definitions of oral diseases. In addition, designing a prospective study to identify the time- course of oral ulcers related to AS would be of interest to identify the temporal association between the two conditions.

### Declaration

This research was conducted using the UK Biobank Resource under Application Number 26307. The authors declare that they have no conflict of interests. HMA received funding from the Higher Committee for Education Development in Iraq (HCED-Iraq) to undertake her PhD. The authors are grateful to Professor Gary Macfarlane (Epidemiology Group, University of Aberdeen) for his valuable comments and suggestions in the analysis and drafting of the manuscript.
